# The Severity of Postoperative Pancreatic Fistula Predicts 30-Day Unplanned Hospital Visit and Readmission after Pancreaticoduodenectomy: A Single-Center Retrospective Cohort Study

**DOI:** 10.3390/healthcare10010126

**Published:** 2022-01-08

**Authors:** Hao-Wei Kou, Chih-Po Hsu, Yi-Fu Chen, Jen-Fu Huang, Shih-Chun Chang, Chao-Wei Lee, Shang-Yu Wang, Chun-Nan Yeh, Ta-Sen Yeh, Tsann-Long Hwang, Jun-Te Hsu

**Affiliations:** 1 Department of Surgery, Division of General Surgery, Linkou Chang Gung Memorial Hospital, Chang Gung University, Taoyuan City 333, Taiwan; jeffreykou0417@gmail.com (H.-W.K.); center061990@gmail.com (Y.-F.C.); b9302071@cgmh.org.tw (S.-C.C.); alanchaoweilee@hotmail.com (C.-W.L.); shangyuwang@gmail.com (S.-Y.W.); yehchunnan@gmail.com (C.-N.Y.); tsy471027@cgmh.org.tw (T.-S.Y.); hwangtl@cgmh.org.tw (T.-L.H.); 2 Department of Surgery, Division of Trauma and Emergency Surgery, Linkou Chang Gung Memorial Hospital, Chang Gung University, Taoyuan City 333, Taiwan; chihpo1227@gmail.com (C.-P.H.); m7626@cgmh.org.tw (J.-F.H.)

**Keywords:** pancreaticoduodenectomy, pancreatic fistula, unplanned hospital visit, readmission

## Abstract

Background: Unplanned hospital visits (UHV) and readmissions after pancreaticoduodenectomy (PD) impact patients’ postoperative recovery and are associated with increased financial burden and morbidity. The aim of this study is to identify predictive factors related to these events and target the potentially preventable UHV and readmissions. Methods: We enrolled 518 patients in this study. Characteristics were compared between patients with or without UHV and readmissions. Results: The unplanned visit and readmission rate was 23.4% and 15.8%, respectively. Postoperative pancreatic fistula (POPF) grade B or C, the presence of postoperative biliary drainage, and reoperation were found to be predictive factors for UHV, whereas POPF grade B or C and the presence of postoperative biliary drainage were independently associated with hospital readmission. The most common reason for readmission was an infection, followed by failure to thrive. The overall mortality rate in the readmission group was 4.9%. Conclusions: UHV and readmissions remain common among patients undergoing PD. Patients with grade B or C POPF assessed during index hospitalization harbor an approximately two-fold increased risk of subsequent unplanned visits or readmissions compared to those with no POPF or biochemical leak. Proper preventive strategies should be adopted for high-risk patients in this population to maintain the continuum of healthcare and improve quality.

## 1. Introduction

Pancreaticoduodenectomy (PD) is surgery for both benign and malignant diseases affecting the periampullary area. It is a complex operation in general surgery owing to the anatomical complexity and mandatory need of multiple anastomoses to restore the alimentary tract, which inevitably increases the rate of morbidity. The reported morbidity rate of PD is as high as 60% [1,2]. Readmission is considered a postoperative complication after PD and is recognized as a quality metric of performance [3,4,5]. To improve patients’ outcomes, investigation of the risk factors associated with this complication has been of great interest [6,7,8,9,10]. Various risk factors have been proven to be associated with readmission after PD, and the presence of postoperative pancreatic fistula (POPF) is one of them. However, none of these studies reported the relationship between the grade of POPF and its impact on hospital readmission [2,9,10,11]. Additionally, the International Study Group for Pancreatic Fistula (ISGPF) had an update on the definition of POPF in 2016, which redefined grade A POPF as a biochemical leak since it had no clinical importance [12]. Collectively, the association between the severity of POPF and hospital readmission remains unclear. Proper risk stratification of these patients could assist physicians in making precise decisions to discharge patients and improve the quality of healthcare [6,13].

In addition to readmission, unplanned hospital visits are also troublesome postoperative events for patients after discharge [14,15,16,17]. The event not only impacts patients’ postoperative recovery and satisfaction but also increases the financial burden and additional resource utilization [3,4,5,7,18]. However, few studies have explored risk factors related to unplanned hospital utilization among patients undergoing PD [7,8,9,10]. Hence, we have conducted a retrospective study of patients undergoing PD to identify predictive factors associated with unplanned hospital visits and/or readmissions within 30 days after discharge. Additionally, we also report the primary reason, management, and outcomes in patients experiencing unplanned hospital visits and readmissions.

## 2. Materials and Methods

### 2.1. Patient Population

We recruited patients undergoing PD (classical or pylorus-preserving) at Linkou Chang Gung Memorial Hospital from October 2011 to July 2018. Patients who were younger than 18 years, received total pancreatectomy, or experienced in-hospital mortality during index hospitalization were excluded. This retrospective analysis was approved by the Institutional Review Board of the Chang Gung Medical Foundation (approval no. 202000980B0), which approved the waiver for informed consent. All methods in this study were carried out in accordance with our institutional guidelines and regulations.

### 2.2. Data Collection and Definition

We retrospectively collected data, including demographic characteristics, preoperative laboratory examinations, operative details, and complications. Patients who had unplanned hospital visits, including emergency departments or outpatient clinics within 30 days after discharge, were defined as the unplanned visit group. Among them, patients who required hospital admission for further treatment were defined as the readmission group. The decision of hospital discharge or readmission was made according to the physician’s experience and clinical judgment. We also reviewed the primary cause, treatment details, and outcomes of patients who experienced unplanned visits or readmissions. Surgical complications were categorized based on the Clavien–Dindo classification. Total psoas muscle area was defined as the cross-sectional area of the psoas muscle at the level of the third lumbar vertebrae and then normalized to the patient’s height (mm^2^/m^2^). Preoperative biliary drainage included percutaneous transhepatic drainage, percutaneous transhepatic gallbladder drainage, or endoscopic retrograde biliary drainage, while the postoperative biliary drainage tubes referred to intraoperative indwelling, a trans-anastomotic drainage tube of hepaticojejunostomy, or preservation of preoperative drainage after surgery. The type of pancreaticojejunostomy reconstruction and the use of a pancreatic stent (external, internal, or none) were based on the surgeon’s decision and preference. The surgeon’s experience was determined by counting the overall number of PD performed previously and was classified into two groups (<50 PDs and ≥50 PDs) [19].

### 2.3. Definition and Management of POPF

We assessed the severity of POPF according to the finding during index hospitalization and classified the POPF grade based on the definition from ISGPF [12]. For patients with grade B POPF, we treated infective sequelae with antibiotics, kept nihil per os, or gave somatostatin analogs. If intra-abdominal peripancreatic fluid collection was detected, radiological percutaneous drainage was arranged if feasible. If there were signs of hemorrhagic complications, we arranged angiographic intervention for embolization. For patients who suffered from postoperative uncontrolled infection or hemorrhage due to POPF, we performed re-laparotomy to treat these complications. For patients with POPF-related organ failure, they were admitted to the surgical intensive care unit for specific organ support techniques such as renal replacement therapy or mechanical ventilation. In cases with organ failure or need of re-laparotomy, patients were classified as grade C POPF.

We routinely placed intraperitoneal drains intraoperatively for monitoring POPF. The intraperitoneal drains normally were removed 7–10 days after surgery if feasible. The decision to remove drains is based on clinical judgments. The application of biliary drainage and pancreatic drainage tubes following PD were based on the surgeon’s preference. If present, the biliary or pancreatic drains were removed in the outpatient clinic after discharge.

### 2.4. Statistical Analysis

Pearson’s chi-squared test or Fisher’s exact test was applied to compare categorical variables, which are presented as frequencies and percentages. The Kolmogorov–Smirnov test was used to check the normality of the continuous variables. For continuous variables, Student’s *t*-test in cases of normal distribution of data or the Mann–Whitney *U* test in cases of the non-normal distribution of data was used for comparison. Data are presented as mean ± standard deviation or median with interquartile ranges (IQRs). Factors that were statistically significant in the univariate analysis were subjected to multivariate logistic regression analysis. Statistical significance was set at *p* < 0.05. All statistical analyses were performed using IBM SPSS Statistics 21 (IBM Corporation, Armonk, NY, USA).

## 3. Results

A total of 518 patients (313 men and 205 women) who underwent PD were enrolled in this study. The mean age of the cohort was 62.1 ± 11.8 years, ranging from 23 to 87 years, and 45.2% of patients were aged ≥ 65 years. Most patients (83.2%) received PD for malignant diseases. The details of the pathological diagnoses are listed in Table 1. Thirty patients (5.8%) underwent concomitant vascular resection; all of them received portal vein resection, and none of our patients had arterial resections. Forty-eight (9.3%) patients underwent additional organ resection along with PD. Among them, 24 patients had colectomy, 23 patients had hepatectomies, 12 patients had total gastrectomies, and 1 patient had a small bowel resection; 10 patients had two additional organ resections, and 1 patient had three additional organ resections. All of our patients reconstructed with pancreaticojejunostomy after PD; the reconstruction methods included 284 duct-to-mucosa techniques (54.8%), 170 invagination techniques (32.8%), 38 Blumgart techniques (7.3%), and 26 unknown techniques (5.0%). In this study, 106 (20.5%) of our patients were discharged with intraperitoneal drains, 186 (35.9%) patients with biliary drainage tubes, and 300 (57.9%) patients with external pancreatic drainage tubes; 358 (69.1%) out of 518 patients had at least one drainage tube left in place at the initial discharge.

Patients were divided into planned visit (*n* = 397, 76.6%) and unplanned visit groups (*n* = 121, 23.4%), or non-readmission (*n* = 436, 84.2%) and readmission groups (*n* = 82, 15.8%). Table 2 and Table 3 compare the clinical demographics of patients, laboratory examinations, operative details, and postoperative complications regarding unplanned visits and hospital readmission. There were no significant differences in preoperative variables, intraoperative variables, and the surgeon’s experience between the planned and unplanned visit groups or between the non-readmission and readmission groups. Compared with the planned visit group, the unplanned visit group had a significantly more severe POPF grade (*p* = 0.006) and a higher rate of presence of the postoperative biliary drainage tube (*p* = 0.001), reoperation (*p* = 0.025), radiological percutaneous drainage (*p* = 0.010), and complication grade > III (*p* = 0.033). Compared with the non-readmission group, greater percentages of the postoperative biliary drainage tube (*p* = 0.032) and higher grades of POPF (*p* = 0.004) were noted in the readmission group. POPF grade B or C (odds ratio (OR) =1.886; confidence interval (CI), 1.202–2.960; *p* = 0.006), the presence of postoperative biliary drainage (OR = 2.140; CI, 1.398–3.278; *p* < 0.001), and reoperation (OR = 4.803; CI, 1.242–18.564; *p* = 0.023) were found to be predictive factors for unplanned hospital visits after multivariate logistic regression analysis, whereas POPF grade B or C (OR = 2.206; CI, 1.361–3.576; *p* = 0.001), and the presence of postoperative biliary drainage (OR = 1.805; CI, 1.111–2.932; *p* = 0.017) were independently associated with hospital readmission (Table 4).

Table 5 lists the primary reasons for unplanned hospital visits and readmissions within 30 days after discharge. Among 121 patients who experienced unplanned hospital visits, 82 (67.8%) patients required readmission for further treatment, while the remaining 39 (32.2%) could be discharged home after management. The most common reason for readmission was infection (34.4%) followed by failure to thrive (26.8%), whereas drainage tube-related problems (35.9%) and gastrointestinal symptoms (33.3%) were the frequent causes in those who could be discharged home after a hospital visit, in no need of readmission. Among 82 patients in need of readmission, 12 (14.6%) developed complications greater than grade III, including four patients undergoing percutaneous drainage for intra-abdominal abscess, three endoscopic hemostasis for gastrointestinal hemorrhage, one pigtail indwelling for pleural effusion, and one angioembolization for pseudoaneurysm; four died due to myocardial infarction (*n* = 2), sepsis (*n* = 1), and cancer cachexia (*n* = 1). The overall mortality rate in the readmission group was 4.9%. 

**Figure 1:**
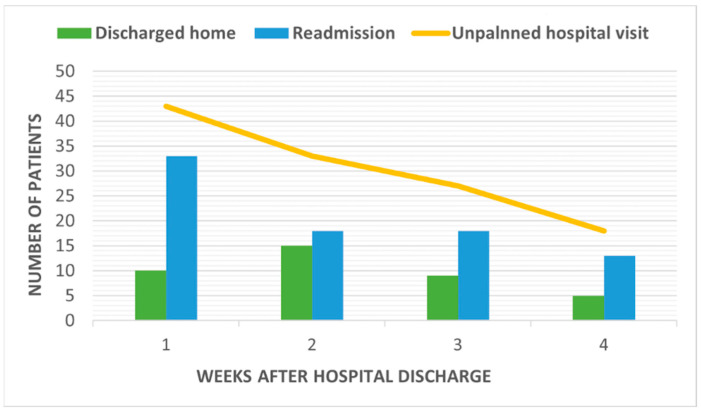
Number of unplanned hospital visits by patients following pancreaticoduodenectomy by week.

Figure 1 depicts the number of unplanned hospital visits and readmissions following PD in terms of weeks. Forty-three patients (52.4%) with unplanned hospital visits were identified within 1 week after discharge, and the number gradually decreased weekly. A similar trend was observed in patients in the readmission group. The median time from first hospital discharge to unplanned visit was 10 days (IQR, 5.5–19 days), and readmission also occurred at a median of 10 days (IQR, 4–19 days) after the first discharge.

## 4. Discussion

Readmission has been considered as an indicator of the quality of healthcare for patients undergoing PD [5]. Recent studies have focused on decreasing the rate of readmission and healthcare resource utilization [5,7,13,18]. In the present study, the readmission rate of patients receiving PD was 15.8%, which is consistent with previous reports, ranging from 14% to 27% [2,3,4,6,8,9,10,11,18,20]. In addition, unplanned hospital utilization after discharge was 23.4% in our series. The clinical importance of unplanned hospital visits has been investigated in the field of postoperative care [14,15,16,17]. Unplanned hospital visits after PD should be considered as a complication or an indicator of the quality of healthcare, just like hospital readmission. Studies have demonstrated that preventive interventions should be implemented in patients undergoing PD who are at a high risk of unplanned hospital utilization after discharge to reduce the incidence of medical events [3,8]. Our present findings suggest that the severity of POPF and the presence of postoperative biliary drainage were independently associated with unplanned hospital visits and readmissions 30 days after discharge, providing valuable information for physicians, policy-makers, administrators, and medical insurers to adopt preventive strategies to improve the quality of healthcare.

POPF has been suggested to be one of the major determinants of morbidity after PD [1,12,21]. In 2016, ISGPF redefined “grade A POPF” into “biochemical leak” due to it being clinically irrelevant, while the clinical importance of grade B and grade C POPF [12,22] were more emphasized. Instead of using the classification of ISGPF in 2016, studies categorized patients into those with or without POPF and reported that POPF was associated with the rate of readmission, which could not fully reflect the impact of POPF severity or grade on readmission [2,9,10,11]. Table 6 summarizes studies indicating POPF as an independent factor of hospital readmission. Our present results showed that POPF was not only an independent predictor of hospital readmission but also of unplanned hospital visits. Additionally, patients with grade B or grade C POPF had an approximately two-fold increased risk of unplanned visits and readmissions compared to those with no POPF or biochemical leak. To the best of our knowledge, our study is the first to provide evidence suggesting a relationship between the grade of POPF and readmission. Therefore, proper management of POPF before discharging the patient may be deemed as a part of a strategy to decrease the rate of unplanned visits or readmissions. Implementation of appropriate measures, such as comprehensive discharge education, coordination of discharge planning, and monitoring by phone call or enhanced follow-up, can keep the continuum of healthcare to reduce the rate of readmission [6,23,24]. Moreover, in cases with grade B or grade C POPF, different levels of preventive interventions should be applied due to their distinct risk of disease. Further studies are needed to confirm whether suitable management can minimize the rate of readmission for these patients.

In addition to POPF, we identified reoperation as an independent risk factor associated with readmission, in line with previous studies [2,9]. In contrast to our results, other authors also found that age, underlying comorbidity, and complexity of the operation predicted readmission [3,7,8,9,10,20]. In addition, we attempted to explore the relationship between readmission and the psoas muscle area, which has been used to diagnose sarcopenia or fragility [25]. No statistical difference was noted between the studied groups in either the unplanned visit group or the readmission group, suggesting that fragility may have less impact on the short-term outcomes. Collectively, our present results indicated that unplanned hospital visits and readmissions were associated with postoperative events rather than preoperative or operative characteristics. The occurrence of postoperative complications during the index hospitalization was a significant risk factor for hospital readmission, as evidenced by our observations and other reports [4,6]. Furthermore, our findings were in line with a meta-analysis conducted by Howard et al., showing that readmission was not associated with patient demographics or comorbidities [7].

In the current study, we discovered that among 121 patients with unplanned hospital visits within 30 days after discharge, drainage tube-related problems were observed in 14 patients in the discharged group and two in the readmission group. Drainage tube-related problems comprising 35.9% of patients in the discharged group (*n* = 39) implied that this problem had relatively low acuity. The role of the routine intraoperative placement of drainage tubes during PD remains controversial [26,27]. Several meta-analysis studies have demonstrated comparable results between patients with or without routine drainage tube placement after PD, particularly for those at low risk of POPF [27,28]. Although it is not feasible to conclude the necessity of drainage tube placement, these results exhibited a notion of avoiding unnecessary drainage tube placement to avert postoperative complications [26,27]. Moreover, early removal of the drainage tube has been recognized as a part of enhanced recovery after surgery protocol for PD [29]. Studies showed that early removal of drains reduced intra-abdominal infection and pancreatic fistula in patients undergoing PD [9,30]. Therefore, patients may not benefit from the prolonged placement of intra-abdominal drainage tubes in low-risk patients with POPF. In the present study, the presence of a biliary drainage tube following PD was an independent predictor of both unplanned visits and readmissions. Furthermore, studies have indicated that preoperative biliary drainage procedures increased postoperative complications in patients undergoing PD [29,31]. Using a diversional drainage tube for biliary-enteric anastomoses following PD to reduce the rate of leakage remains unclear [32]. Only a few studies with small sample sizes have reported the benefit of biliary drainage following PD [33,34,35]; thus, the beneficial effects or necessity of biliary drainage after PD should be further confirmed by a large-scale prospective randomized trial. Collectively, we suggest that minimizing the use of biliary drainage tubes after PD or early removal of drainage tubes during the index hospitalization might be considered as a potential way to minimize the rate of unplanned hospital visits and readmissions. 

There are some limitations to the present study. First, this was a single-institution retrospective study over the past decade, in which selection bias could not be overlooked. Second, we cannot completely acquire the data of patients who seek medical help after discharge in other healthcare facilities. Third, there was no standardization of the use of biliary or pancreatic stenting, placement of preoperative biliary drainage, or the timing of drain removal.

## 5. Conclusions

Unplanned hospital visits and readmissions remain common among patients undergoing PD. Our study indicated that the severity of POPF and the presence of postoperative biliary drainage assessed during index hospitalization independently predicted unplanned hospital visits and readmissions within 30 days after discharge. Accurate stratification of high-risk populations can assist physicians in adopting proper preventive strategies and making decisions carefully while discharging patients in order to improve the quality of healthcare.

## Figures and Tables

**Table 1:** Pathological diagnosis of resection lesions (*n* = 518).

Diagnosis	Number of Patients (%)
**Malignant neoplasm**	
Pancreatic carcinoma	181 (34.9)
Ampullary carcinoma	124 (23.9)
Biliary tract carcinoma	57 (11.0)
Gastric carcinoma	38 (7.3)
Duodenal carcinoma	25 (4.8)
Colon carcinoma	5 (1.0)
Retroperitoneal carcinoma	1 (0.2)
**Benign or borderline neoplasm**	
Cystadenoma/adenoma	22 (4.2)
Pancreatic neuroendocrine tumor	7 (1.4)
Low or non-risk gastrointestinal stromal tumor	8 (1.5)
Intraductal papillary mucinous neoplasm	7 (1.4)
Choledochal cyst	4 (0.8)
Solid-pseudopapillary neoplasm	2 (0.4)
Other lesions	3 (0.6)
**Non-neoplastic benign disease**	
Pancreatitis	26 (5.0)
Biliary tract Inflammation/Infection	6 (1.2)
Others	2 (0.4)

**Table 2:** Clinical demographics of patients categorized by unplanned hospital visit or readmission.

Variables		Planned Visit Group (*n* = 397)	Unplanned Visit Group (*n* = 121)	*p* Value	Non-Readmission Group (*n* = 436)	Readmission Group (*n* = 82)	*p* Value
**Male**		235 (59.2)	78 (64.5)	0.299	265 (60.8)	48 (58.5)	0.703
**Age (years)**		63 (54–71)	64 (54.5–71)	0.395	63 (54–71)	65.5 (54.8–74)	0.101
**Age ≥ 65 years**		175 (44.1)	59 (48.8)	0.365	190 (43.6)	44 (53.7)	0.092
**Body mass index (kg/m^2^)**		22.8 (20.7–25.5)	22.8 (21.3–24.7)	0.666	22.8 (20.6–25.3)	22.9 (22.0–25.1)	0.235
**Malignant disease**		332 (83.6)	99 (81.8)	0.641	363 (83.3)	68 (82.9)	0.942
**History of abdominal surgery**		13 (3.3)	6 (5.0)	0.409	15 (3.4)	4 (4.9)	0.521
**Serum bilirubin level (mg/dL)**		1.4 (0.6–3.9)	1.1 (0.5–3.4)	0.449	1.3 (0.5–3.9)	1.2 (0.5–3.1)	0.491
**Serum albumin level (g/dL)**		3.87 (3.51–4.23)	3.81 (3.47–4.17)	0.856	3.87 (3.50–4.23)	3.79 (3.48–4.13)	0.890
**Serum Hemoglobin level (g/dL)**		12.0 (10.6–13.4)	12.0 (10.6–13.6)	0.660	12 (10.7–13.5)	11.9 (10.5–13.6)	0.559
**Preoperative biliary drainage**		158 (39.8)	51 (42.1)	0.645	172 (39.4)	37 (45.1)	0.337
**Total psoas muscle area** **(mm^2^/m^2^)**		515.3 (389.8–618.0)	495.7 (394.4–652.0)	0.837	510.8 (390.8–619.9)	491.9 (387.8–619.7)	0.751
**ASA**	1	5 (1.3)	1 (0.8)	0.862	5 (1.1)	1 (1.2)	0.862
	2	70 (17.6)	20 (16.5)		77 (17.7)	13 (15.9)	
	3	319 (80.4)	100 (82.6)		351 (80.5)	68 (82.9)	
	4	3 (0.8)	0 (0.0)		3 (0.7)	0 (0.0)	
**CCI**	≤2	59 (14.9)	16 (13.2)	0.806	64 (14.7)	11 (13.4)	0.924
	3–4	136 (34.3)	45 (37.2)		151 (34.6)	30 (36.6)	
	≥5	202 (50.9)	60 (49.6)		221 (50.7)	41 (50.0)	

ASA, American Society of Anesthesiologists physical status classification; CCI, Charlson Comorbidity Index. Continuous data are expressed as median (interquartile range), and categorical data are expressed as a number (%).

**Table 3:** Operative details and postoperative events during index hospitalization of patients categorized by unplanned hospital visit or readmission.

Variables		Planned Visit Group (*n* = 397)	Unplanned Visit Group (*n* = 121)	*p* Value	Non-Readmission Group (*n* = 436)	Readmission Group (*n* = 82)	*p* Value
**Operative method**	Classical PD	258 (65.0)	79 (65.3)	0.951	289 (66.3)	48 (58.5)	0.177
	PPPD	139 (35.0)	42 (34.7)		147 (33.7)	34 (41.5)	
**Operative time (minutes)**		479 (401–572)	503 (421–604)	0.070	483 (403–575)	493 (401–603)	0.376
**Surgeon Experience** *	<50 PDs	203 (51.1)	64 (52.9)	0.735	225 (51.6)	42 (51.2)	0.949
	≥50 PDs	194 (48.9)	57 (47.1)		211 (48.4)	40 (48.8)	
**Blood loss (mL)**		350 (200–625)	400 (200–825)	0.118	350 (200–650)	400 (200–813)	0.393
**Combine organ resection**		37 (9.3)	11 (9.1)	0.877	40 (9.2)	8 (9.8)	0.945
**Portal vein resection**		23 (5.8)	7 (5.8)	0.997	24 (5.5)	6 (7.3)	0.451
**Postoperative biliary** **drainage tube**		127 (32.0)	59 (48.8)	0.001	148 (33.9)	38 (46.3)	0.032
**Pancreatic stent**		314 (84.6)	104 (89.7)	0.176	347 (85.3)	71 (88.8)	0.413
**POPF**	No or BL	271 (68.3)	65 (53.7)	0.006	295 (67.7)	41 (50.0)	0.004
	Grade B	122 (30.7)	52 (43.0)		136 (31.2)	38 (46.3)	
	Grade C	4 (1.0)	4 (3.3)		5 (1.1)	3 (3.7)	
**Reoperation**		7 (1.8)	7 (5.8)	0.025	11 (2.5)	3 (3.7)	0.473
**Angioembolization**		4 (1.0)	3 (2.5)	0.363	6 (1.4)	1 (1.2)	1.000
**Radiological percutaneous drainage**		22 (5.5)	15 (12.4)	0.010	30 (6.9)	7 (8.5)	0.593
**Complication grade ≥ III**		51 (12.8)	25 (20.7)	0.033	64 (14.7)	12 (14.6)	0.992
**Length of hospital stay (days)**		21 (16–30)	22 (17–31.5)	0.159	21 (16–31)	22 (18–30.25)	0.365

PD, pancreaticoduodenectomy; PPPD, pylorus-preserving pancreaticoduodenectomy; POPF, postoperative pancreatic fistula; BL, biochemical leak. Continuous data are expressed as median (interquartile range), and categorical data are expressed as a number (%). *, the surgeon’s experience was determined by counting the overall number of PDs performed previously [19].

**Table 4:** Multivariate logistic regression analysis of factors for unplanned hospital visits or readmission.

Patient Cohort	Variables	Odds Ratio	95% CI	*p* Value
Unplanned visit group	POPF grade (Grade B or C vs. No or BL)	1.886	1.202–2.960	0.006
	Postoperative biliary drainage (Yes vs. No)	2.140	1.398–3.278	<0.001
	Reoperation (Yes vs. No)	4.803	1.242–18.564	0.023
	Radiological percutaneous drainage (Yes vs. No)	2.306	0.846–6.287	0.103
	Complication grade ≥ III (Yes vs. No)	0.728	0.305–1.739	0.475
Readmission group	POPF grade (Grade B or C vs. No or BL)	2.206	1.361–3.576	0.001
	Postoperative biliary drainage (Yes vs. No)	1.805	1.111–2.932	0.017

CI, confidence interval; POPF, postoperative pancreatic fistula; BL, biochemical leak.

**Table 5:** Primary reasons for unplanned hospital visits within 30 days after discharge (*n* = 121).

Discharged Home (*n* = 39)		Readmission (*n* = 82)	
**Infection**		**Infection**	
Intraabdominal infection	2 (5.1)	Intraabdominal infection	28 (34.1)
Wound infection	3 (7.7)	Wound infection	11 (13.4)
**Gastrointestinal symptoms**		Pneumonia	2 (2.4)
Abdominal discomfort	10 (25.6)	Urinary tract infection	1 (1.2)
Nausea/Vomiting	3 (7.7)	Other infection	1 (1.2)
**Drainage tube related problems**	14 (35.9)	**Failure to thrive** **^1^**	22 (26.8)
**Other symptoms**	7 (17.9)	**Gastrointestinal symptoms**	
		Ileus	5 (6.1)
		Gastrointestinal bleeding	4 (4.9)
		**Vascular events**	
		Myocardial infraction	2 (2.4)
		Stroke	1 (1.2)
		GDA pseudoaneurysm	1 (1.2)
		**Drainage tube related problems**	2 (2.4)
		**Other symptoms**	2 (2.4)

^1^ Including dehydration, malnutrition, and electrolyte imbalance. Gastroduodenal artery, GDA. Data are expressed as a number (%).

**Table 6:** Studies indicating POPF as an independent factor of hospital readmission.

Author, Year	Case No.	30-Day Readmission Rate	Variables of POPF	Definition of POPF	OR	95% CI
Ahmad, 2012 [10]	1302	15%	With vs. Without	ISGPF (2005)	2.4	1.2–4.8
Fong, 2014 [2]	1173	16%	With vs. Without	ISGPF (2005)	1.86	1.220–2.834
Mosquera, 2016 [11]	220	26.8%	With vs. Without	ISGPF (2005)	4.55	1.3–16.3
Ramanathan, 2018 [9]	18440	18.7%	With vs. Without	Clinical diagnosis or persistent drainage with specific condition ^a^	1.64	NA
Kou, 2021 (Current study)	518	15.8%	Grade B/C vs. No POPF/BL	ISGPF (2016)	2.206	1.361–3.576

POPF, postoperative pancreatic fistula; OR, odds ratio; CI, confidence interval; ISGPF, International Study Group for Pancreatic Fistula; NA, not available; BL, biochemical leak. ^a^ with one of the following conditions: nihil per os and parenteral nutrition, drain continued beyond 7 days, percutaneous drainage, reoperation, or spontaneous wound drainage.

## Data Availability

These data are not publicly available.
